# Vulvar extramammary Paget’s disease with clitoral involvement: case series highlighting the role of topical treatment

**DOI:** 10.1097/JW9.0000000000000238

**Published:** 2026-02-11

**Authors:** Johanny Lopez Dominguez, Nour Kibbi

**Affiliations:** a Department of Dermatology, Stanford Medicine, Redwood City, California

**Keywords:** extramammary Paget’s disease, Mohs micrographic surgery, topical treatment, vulvar extramammary paget’s disease

**What is known about this subject in regard to women and their families?**

- Vulvar Extramammary Paget’s Disease (EMPD) with clitoral hood involvement can cause significant morbidity for patients. Historically, vulvar EMPD has been treated with radical or skinning vulvectomy, which can have high morbidity for women.

**What is new from this article as messages for women and their families?**

- In patients with vulvar EMPD with clitoral involvement, imiquimod can be considered as an alternative treatment modality for disease control with good tolerability and minimal morbidity.

Extramammary Paget’s disease (EMPD) is a rare, highly recurrent malignant neoplasm arising in anogenital skin. The risk of secondary malignancy and rate of invasive disease differ by anatomic subtype.^1,2^ Historically, vulvar EMPD has been treated with radical or skinning vulvectomy. Mohs micrographic surgery (MMS) is a tissue-sparing technique that can minimize surgical morbidity while offering surgical cure, making it the gold standard treatment. It is unknown how often vulvar EMPD involves functionally important structures such as the clitoris or urethral meatus, yet surgical intervention can be morbid, and alternative modalities have been used with mixed success.^3^ Here, we present our experience with 2 cases of EMPD with clitoral involvement treated successfully with imiquimod cream.

## Case 1

A 36-year-old female presented with a 3-year history of refractory erosions of the right labium majus, mons pubis, and clitoris. Her symptoms included pruritus and dyspareunia. Given lack of improvement with topical antifungals, a biopsy was performed and confirmed EMPD. She was referred to our clinic for MMS. To assess the extent of disease, scouting biopsies were performed, confirming intraepidermal disease with involvement of the clitoris. Malignancy workup was negative. During her consultation, she indicated a desire to maintain sexual and reproductive function. Since surgical resection would result in functional impairment, she was treated with imiquimod 5% cream 2 to 3 times weekly and completed 53 applications over the course of 8 months with multiple pauses in treatment due to imiquimod-related skin breakdown. While off imiquimod, she was able to maintain sexual function. The patient achieved clinical remission and is being monitored closely for recurrence.

## Case 2

A 70-year-old female presented with an 8-year history of an asymptomatic, focal erosion on the right labium majus. The rash was treated as presumed lichen sclerosus but did not respond to clobetasol ointment. Given the lack of improvement, a biopsy was performed, confirming EMPD. The patient was referred to our clinic for MMS. During her consultation, scouting biopsies were performed, confirming intraepidermal disease with clitoral involvement. Malignancy workup was negative. The patient had significant pain following the biopsies making her reluctant to undergo surgical intervention. Because her EMPD was asymptomatic, active surveillance vs noninvasive modalities were discussed. The patient elected to trial imiquimod 2 times weekly and completed 14 applications with an interval resolution of erosions of the right labium majus. She is being monitored for recurrence and is open to retreatment.

Patients with vulvar EMPD require special considerations with an individualized approach when planning treatment options. MMS is the gold standard for treatment, offering the highest cure rate. In more extensive cases, it is also important to collaborate with gynecology colleagues for full discussion of surgical approaches. In some cases, surgical management can have high morbidity given involvement of critical structures^2^ and patients may prefer a nonsurgical approach despite lower cure rates. During surgical consultation, scouting biopsies are essential to fully map the disease extent and depth of invasion. For intraepidermal EMPD with clitoral involvement, weighing the options is critical: patients who desire to preserve sexual function may benefit from conservative management. Beyond sexual impact, surgical intervention in this area can pose an unacceptable psychological burden. In patients with intraepidermal vulvar EMPD with clitoral involvement, imiquimod can be considered as an alternative treatment modality for disease control with good tolerability and minimal morbidity. Given EMPD is highly recurrent, frequent follow-up to assess response during treatment and to monitor for recurrence after treatment is essential.

## Conflicts of interest

None.

## Funding

None.

## Study Approval

N/A

## Author contributions

JLD: Participated in research design, writing of the paper, and performance of the research. NK: Participated in research design, writing of the paper, and performance of the research.
